# Anatomical References for the Study of 3D Facial Photographs: A Scoping Review and Proposed Preliminary Framework for Comprehensive Facial Evaluation

**DOI:** 10.3390/jcm15156091

**Published:** 2026-08-05

**Authors:** Gonzalo Muñoz, Leonardo Brito, Josefa Alarcon-Apablaza, Antônio Carlos de Oliveira Ruellas, Victor Ravelo, Sergio Olate

**Affiliations:** 1 Department of Pediatric Dentistry and Orthodontics, Faculty of Dentistry, Universidad de La Frontera, Temuco 4811230, Chile; 2 Undergraduate Research Group in Dentistry (GIPO), Universidad Autónoma de Chile, Temuco 4810101, Chile; leonardo.brito@uautonoma.cl; 3 Program in Morphological Sciences, Faculty of Medicine, Universidad de La Frontera, Temuco 4811230, Chile; josefa.alarcon@ufrontera.cl (J.A.-A.); victor.ravelo.s@gmail.com (V.R.); 4 Department of Pediatric Dentistry and Orthodontics, School of Dentistry, Federal University of Rio de Janeiro, Rio de Janeiro 21941-901, Brazil; aruellas@umich.edu; 5 Center of Excellence in Morphological and Surgical Studies (CEMyQ), Universidad de La Frontera, Temuco 4811230, Chile; sergio.olate@ufrontera.cl; 6 Department of Oral Diagnosis, Division of Oral and Maxillofacial Surgery, State University of Campinas, Piracicaba 13414-903, Brazil

**Keywords:** three-dimensional facial photography, maxillofacial surgery, orthodontics, morphological analysis, scoping review

## Abstract

**Background/Objectives**: Facial deformities compromise craniofacial aesthetics and functionality. Maxillofacial surgery and orthodontics require meticulous planning utilizing tomography and clinical photography. Although tomography provides precise information about bony structures, it has limitations in evaluating soft tissues. Three-dimensional photography emerges as a complementary alternative; however, there is limited information regarding morphological facial analysis in three dimensional (3D) images. **Methods**: A scoping review was conducted according to PRISMA-ScR guidelines and the Joanna Briggs Institute methodological framework. The MEDLINE (PubMed), Scopus, SciELO, and Web of Science databases were searched for studies published between 2017 and 2025 evaluating the use of 3D facial photography in orthodontics, orthognathic surgery, maxillofacial surgery, and related craniofacial applications. Two independent reviewers performed study selection and data extraction, with disagreements resolved by a third reviewer. Anatomical landmarks and facial measurements were descriptively synthesized according to facial regions and measurement type. **Results**: Out of 79 initially identified articles, 18 observational studies examining 3D facial photographs were included. Consequently, the “3D FullFace A” tool was developed, incorporating 13 midline points and 12 bilateral points, enabling 18 linear and 28 angular measurements for comprehensive facial analysis. **Conclusions**: Available evidence suggests that 3D facial photography is a promising tool for orthognathic surgical planning. Key advances identified include reliable stereophotogrammetric landmark identification, quantification of soft-tissue changes following orthognathic and orthodontic treatment, and characterization of facial asymmetry and proportionality.

## 1. Introduction

Facial deformities encompass a range of alterations that impact the aesthetics and functionality of the craniofacial complex. These can range from minor asymmetries and discrepancies [1] to significant malformations caused by various conditions and/or pathologies [2,3,4,5,6,7]. Maxillofacial surgery and orthodontics, alongside other dental specialties, have proven reliable alternatives for restoring function and aesthetics to individuals with such anomalies [2,3,4,7,8]. The execution of these treatments requires detailed planning of each movement, evaluated through a set of complementary examinations, including cone beam computed tomography (CBCT) and clinical photography. [9,10,11,12].

Despite these advantages, such exams yield a limited soft-tissue contrast and representation, making them insufficient for detailed evaluation of skin and superficial muscular structures such as muscles and skin [13,14]. Due to these limitations, clinical photography presents an alternative for such analyses, although the literature contains scant information regarding facial morphology in 3D photographic images. According to Olate et al. [15], most analyses of this type fall into the error of combining 2D and 3D parameters, generating several biases and errors in data extrapolation. Moreover, there is no evidence in the literature that allows for reliable planning of expected outcomes in maxillofacial surgery using 3D images [15,16].

The importance of planning in maxillofacial surgery transcends the acquisition of favorable outcomes. The emotional condition of the patient in the pre-surgical phase and post-operative self-perception issues are among the chief psychological disorders arising from orthognathic surgery, where objective communication can assist in these processes. In this regard, prior knowledge of potential outcomes allows for more appropriate adjustment of expectations, improving pre-surgical anxiety levels and post-surgical result acceptance [17,18,19].

Currently, gender-affirming facial surgeries have transformed the paradigms that traditionally guide surgical planning, demanding greater anatomical knowledge and extensive planning. While planning is based on hard tissues, individuals’ expectations are associated with the expression of these changes in soft tissues [20,21]. Despite these considerations, there is currently no comprehensive 3D facial soft tissue analysis that can serve as a basis for performing such procedures, which constitutes the main limitation in employing and studying these tools.

Therefore, the objective of this study is to conduct a scoping review of facial measurements performed in 3D photographs to synthesize the available evidence on anatomical landmarks and measurement protocols used in 3D facial photography, and to propose a preliminary structured framework for 3D facial image analysis as a foundation for future validation research. This approach aims to yield objective and reproducible information about facial morphology applicable to both general and detailed facial analysis.

## 2. Materials and Methods

### 2.1. Search Strategy

A scoping review was conducted to identify and map the anatomical landmarks and measurements used in three-dimensional (3D) facial photography for facial morphological analysis in orthodontics, orthognathic surgery, and maxillofacial surgery. The review methodology was developed according to the Preferred Reporting Items for Systematic Reviews and Meta-Analyses extension for Scoping Reviews (PRISMA-ScR) guidelines [22]. The objective of the review was to characterize the available evidence regarding facial reference points, linear measurements, angular measurements, and analytical methods used in 3D facial photographs, to support the development of a comprehensive protocol for facial morphological evaluation.

This study was conducted in accordance with the ethical principles established by the Declaration of Helsinki and applicable national regulations (Laws No. 20.120, No. 19.628, and No. 20.584). The research protocol was reviewed and approved by the Scientific Ethics Committee of Universidad de La Frontera, Temuco, Chile (Act No. 110_23, Protocol UFRO No. 075/23, approved on 9 August 2023). Written informed consent was obtained from all participants prior to their inclusion in the study. The facial images presented in this article were obtained with the explicit written consent of the participant for their use in academic publications.

Accordingly, the review aimed to answer the following question: “What anatomical reference points and facial measurements have been used in the analysis of 3D facial photographs?”.

A comprehensive literature search was conducted in the MEDLINE, Scopus, Web of Science, and SciELO databases to identify studies reporting the use of 3D facial photography for facial morphological analysis. The terms selected for the search were those previously employed by Muñoz et al. [23]. The keywords were combined utilizing the Boolean operators OR and AND. The electronic search included studies published between January 2006 and June 2026. Additionally, a manual search was performed by screening the reference lists of the included articles to identify potentially relevant studies not retrieved during the electronic search process.

Although the primary electronic search was restricted to studies published between January 2017 and June 2025, studies published within the last 20 years (2005–2025) were considered eligible for inclusion when reporting foundational anatomical landmark definitions. This broader temporal scope was justified by the cumulative nature of three-dimensional facial morphology research, in which anatomical reference points represent stable, consensus-based definitions that have been systematically described and refined over several decades. Unlike clinical intervention studies, where methodological advances may rapidly render earlier findings obsolete, anatomical landmark research builds incrementally upon prior work, making temporal exclusion criteria less appropriate as a quality filter.

### 2.2. The Following Search Equation Was Utilized

(((orthognathic surgery 3D AND (fft[Filter])) OR (“orthognathic surgery” AND (fft[Filter]))) OR (“FACIAL SURGERY” AND (fft[Filter]))) OR (“maxilla-facial surgery” AND (fft[Filter]))) AND (“3D photography” AND ((fft[Filter]) AND (2017:2025[pdat])))) OR ((“3D photogrammetry” AND (fft[Filter])) OR (“Imaging, Three-Dimensional/methods”[MAJR] AND ((fft[Filter]) AND (2017:2025[pdat]))) AND ((fft[Filter]) AND (2017:2025[pdat]))) Filters: Full text.

### 2.3. Eligibility Criteria

Studies evaluating the use of 3D facial photography for facial morphological analysis in orthodontics, orthognathic surgery, maxillofacial surgery, or related craniofacial applications were considered eligible for inclusion. Observational studies, clinical studies, case series, and methodological studies reporting anatomical landmarks, facial measurements, or analytical protocols derived from 3D facial images were included. Articles published in English or Spanish and available in full text were considered without geographic restrictions.

Studies based exclusively on 2D photographic analysis, studies performed on animals or non-human models, conference abstracts, editorials, narrative reviews, systematic reviews, and studies lacking sufficient methodological description regarding facial measurements or anatomical reference points were excluded.

### 2.4. Article Selection and Data Extraction

All retrieved records were exported to reference management software (Mendeley 2.90.0; Elsevier, London, UK), and duplicate studies were removed prior to screening. Two independent reviewers (GM and LB) performed the study selection process in two phases. First, titles and abstracts were screened according to the eligibility criteria. Subsequently, full-text evaluation was conducted for potentially relevant studies to confirm their inclusion in the review. Disagreements between reviewers during the selection process were resolved through discussion with a third reviewer (SO). Systematic reviews and narrative reviews retrieved during screening were excluded from formal inclusion per the eligibility criteria; however, their reference lists were screened for backward citation searching to identify primary studies not captured by the electronic search.

Data extraction was independently performed by the same two reviewers using a standardized charting form specifically developed for this review. The extracted information included authors, year of publication, study design, clinical application, imaging system or software utilized, anatomical landmarks, facial measurements, and methodological characteristics related to 3D facial analysis. The identified landmarks and measurements were subsequently categorized according to facial anatomical regions and measurement type (linear, angular, or volumetric) to facilitate evidence synthesis and support the development of the proposed comprehensive facial analysis protocol.

The review aimed to map and characterize anatomical landmarks, facial measurements, and analytical methods used in three-dimensional facial photography rather than evaluate intervention outcomes. Additionally, the heterogeneity of imaging protocols, measurement techniques, and study designs limited the applicability of standardized appraisal tools.

## 3. Results

### 3.1. Study Selection

**Figure 1:**
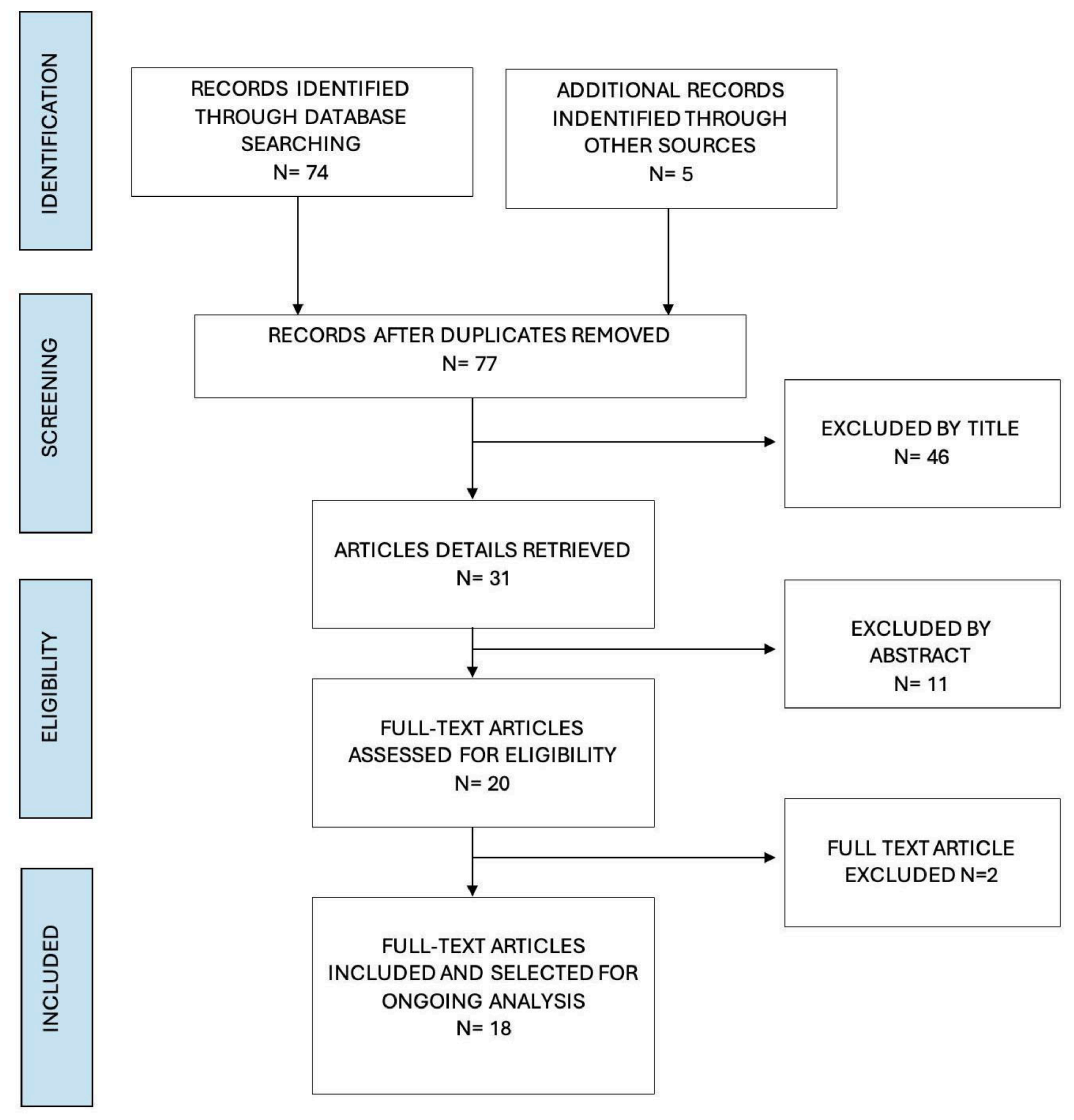
Flow chart for study selection.

A total of 74 articles were identified through the database searches. Subsequently, five were included from the manual search, totaling 79 articles. After removing duplicates, 77 articles remained that met the criteria for inclusion in this analysis. The search and selection process is outlined in Figure 1.

After the initial title screening, 46 articles were excluded due to unrelated topics. Subsequently, 11 studies were eliminated based on abstract reviews, with eight unrelated to the study topic and three being systematic reviews. After full-text evaluations (20 articles), two articles were excluded that referenced 3D measurements but completed their methodology using 2D photographs. Ultimately, 18 articles [20,21,22,23,24,25,26,27,28,29,30,31,32,33,34,35,36,37] corresponding to observational studies analyzing 3D facial photographs in facial, orthognathic, and/or maxillofacial surgery, alongside those using 3D analysis of specific facial structures, were included, as shown in Table 1.

### 3.2. Characteristics of the Selected Studies

**Figure 2:**
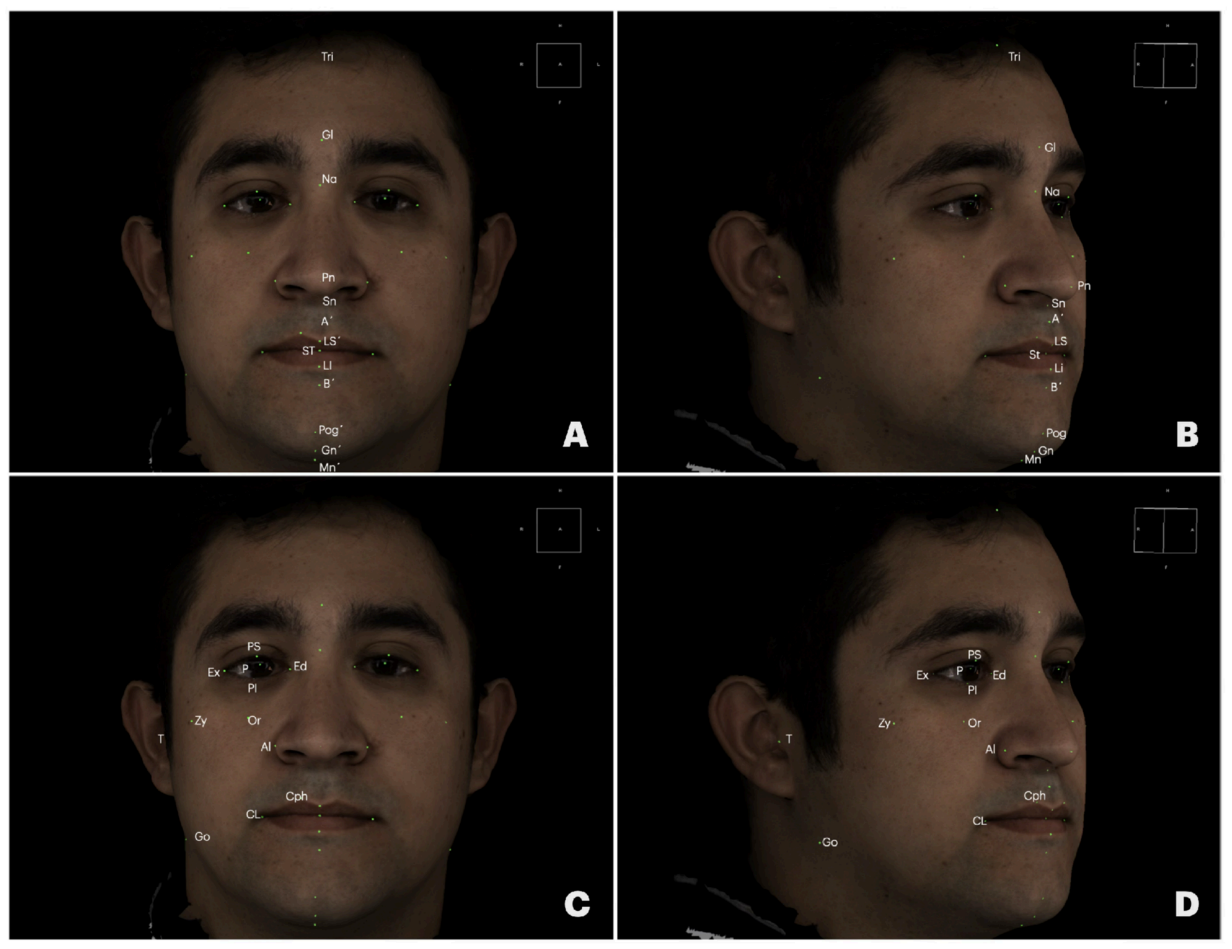
Landmarks. Facial soft-tissue landmarks identified by three-dimensional stereophotogrammetry. (**A**,**B**) Frontal and three-quarter views showing the 13 midline landmarks: trichion (Tri), glabella (Gl), nasion (Na), pronasale (Pn), subnasale (Sn), soft-tissue A-point (A′), labiale superius (LS), stomion (St), labiale inferius (LI), soft-tissue B-point (B′), soft-tissue pogonion (Pog′), gnathion (Gn), and menton (Mn). (**C**,**D**) Frontal and three-quarter views depicting the 12 bilateral landmarks assessed on both sides of the face: zygion (Zy), gonion (Go), endocanthion (Ed), exocanthion (Ex), palpebrale superius (PS), palpebrale inferius (PI), orbitale (Or), pupillare (P), tragion (T), alar base (Al), labial commissure (CL), and crista philtri (Cph). Green dots indicate the digitized landmark positions. Complete definitions and anatomical descriptions of each landmark are provided in Supplementary Tables S1 and S2.

The primary characteristic defining the selected articles was their analysis of 3D facial photographs, either entirely or partially, through studying specific facial structures and developing methodologies to mark points and undertake measurements for morphological evaluation. These described points and measurements from the articles are summarized in Table 2 and Figure 2.

### 3.3. Analysis of Morphological Studies

Most selected studies employed classic anatomical landmarks while also adding various points specified by each author concerning unique facial structures. Thus, a series of points were selected from prior morphological studies, primarily situated in the middle and lower third of the face.

In the middle third, for eye bulb measurements, the edges and center of the eyeball in normal eyelid opening were selected as basic structures for conducting measurements. Consequently, the following points were selected: Ed: Endocanthion; Ex: Exocanthion; PS: Superior Palpebral; PI: Inferior Palpebral; Or: Infraorbital; P: Pupil.

For the nose, the point Na was considered as the most cephalic point of the nasal body, and the subnasal point was regarded as the most caudal point of the same. Additionally, some lateral points were added to evaluate the horizontal component of the nasal body (Points Na: Nasion; Pn: Pronasal; Sn: Subnasal; Al: Right and Left Alar).

In the lower third, reference points were mainly selected on the lips and chin, considering the points of Superior and Inferior Labial through the midline and the points of Labial Commissure and Philtrum Crest bilaterally in the case of the lips, and the points Pogonion, Menton, and Gnathion concerning the chin.

### 3.4. Proposed Measurements for Comprehensive Facial Analysis

Based on the results obtained from the scoping review, a comprehensive facial analysis tool was developed, which includes the points and measurements utilized from the selected articles, referred to as “3D FullFace A,” which we propose as part of this review.

As described in Table 3 and Table 4, from the selection of these points, 18 linear facial measurements and 28 angular measurements were performed to analyze the facial characteristics of each individual.

### 3.5. Recommended Technical Requirements for 3D FullFace A Protocol Implementation

To ensure the reproducibility and comparability of measurements obtained with the 3D FullFace A framework, the following minimum technical conditions are recommended, based on best practices reported across the included studies [23,28,36,38,40,41]:

1. Patient positioning: Frankfurt horizontal plane aligned parallel to the floor; natural head position (NHP) standardized by having the patient look at a fixed point at eye level in front of a mirror prior to capture.
2. Facial expression: neutral, habitual lip seal, teeth in intercuspal occlusion, facial muscles at rest.
3. Imaging system: validated stereophotogrammetry systems (e.g., 3dMD Face System, Bellus 3D) or structured-light scanners with demonstrated geometric accuracy; smartphone-based 3D cameras with verified accuracy may be used as low-cost alternatives pending site-specific validation [42].
4. Lighting conditions: standardized diffuse lighting, avoiding direct lateral light sources that could generate shadows distorting surface geometry.
5. Hair and accessories: hair tied back, earrings removed, glasses removed prior to capture to avoid occlusion of facial landmarks.
6. Coordinate system: Cartesian XYZ with origin at nasion, with X (transverse), Y (anteroposterior), and Z (vertical) axes, consistent with the reference studies included in this review.
7. Landmark digitization software: any 3D mesh analysis software capable of manual landmark digitization and Euclidean distance/angle computation (e.g., Dolphin 3D, Mimics, Meshlab, or equivalent). Digitization should be performed by a trained operator; a minimum of two independent operators is recommended to enable inter-rater reliability assessment.

A full photographic acquisition protocol detailing these parameters has been previously described by Muñoz et al. [23] and serves as the recommended reference for acquisition standardization when implementing the 3D FullFace A framework.

## 4. Discussion

Structural changes during orthognathic and facial surgery impact both hard and soft tissues and must be evaluated over time [24,27,28]. From this perspective, it is interesting to note the assertions made by Olate et al. [15,16], who indicated that there are no available tools that allow proper correlation between soft and hard tissues in orthognathic surgery and outlined several limitations that need to be addressed for the development of such tools [15,16].

Previous studies [16,23] have indicated that precision in point selection and structure evaluation is critical when assessing the reliability of 3D measurements [17,23]. For this reason, developing a facial analysis tool with precisely established and described points, based on widely known prior studies, becomes fundamental for the future of planning and evaluating outcomes in maxillofacial surgery.

Traditionally, precision and reliability analyses were generated through the overlap of hard and soft tissues acquired in both 2D [8,9] and 3D [10,11,12,13], as well as an assessment of clinical parameters. This type of analysis largely requires high-level technical implementation and time investment from both clinicians and patients, being the gold standard for image acquisition and processing based on high-value software and hardware, such as Dolphin 3D Imaging, Vultus 3D, and 3DMD [17,29,38,39].

The advent and development of new technologies, as well as artificial intelligence (AI), allows for the current availability of highly reproducible and reliable tools at lower costs, as seen in products like Bellus 3D Face and the 3D cameras of modern smartphones [23]. Evidence suggests that these tools can capture anatomical details with high resolution and reproducibility. Similar scenarios have been presented by D’Ettorre et al., who exhibited the high reliability displayed by lower-cost tools [42]. This precision is crucial for clinical outcomes, especially in complex procedures like orthognathic surgery, where small variations in measurements can significantly affect clinical results, now achievable with less expensive and more accessible equipment and software [17].

Despite the high quality of 3D images captured by both more costly and complex devices, like 3DMD, and those with lower cost and complexity, such as smartphones, the analysis of these images remains limited, lacking specific protocols for this purpose. In this context, our proposed analysis addresses the limitations previously posed by Olate et al. [15,16], who highlighted the overlap of CBCT 3D parameters with 2D parameters from conventional photographs as a weakness in facial analysis [16,17]. From this perspective, the methodological implementation of the “3D Full Face” analysis represents a significant advancement applicable in both initial facial morphological classification and in technical–surgical planning for various subjects, potentially enabling the future development of clinical tools that allow more precise outcome predictions compared to current methods.

The use of 3D images has previously been explored for simulating surgical results and educating patients about potential aesthetic and functional changes [1,5,8,9,14,15,16,17]. Several authors [17,18,19] demonstrate that the ability to foresee and communicate these results greatly contributes to patient satisfaction and the overall success of treatment [17,18,19]. Considering the high demand for aesthetic procedures and the morphofunctional changes associated with such interventions, the application of a more accurate preoperative diagnosis allows for better patient fidelity and a more objective analysis of the features that determine surgical needs, aligning technical objectives with patients’ expectations. This work represents an initial point for defining future parameters of facial morphology normality or abnormality by incorporating other variables such as age, gender, ethnicity, and additional factors that further complicate facial analyses.

Although 3D photographs offer numerous advantages, they also face technical and methodological challenges. In a previous study, Muñoz et al. [23] proposed standardizing capture techniques and parameters as a starting point for developing appropriate facial analyses [23]. Furthermore, some authors indicate that analyzing these images is crucial for minimizing errors and ensuring the reproducibility of results [36,37,38,39], which supports and sustains the development of these types of facial analysis tools.

Additionally, the direct correlation of 3D facial images with tomographic data may still be limited in some instances, warranting caution in their direct application to planning, emphasizing the necessity to integrate multiple imaging modalities for a comprehensive and accurate evaluation, and highlighting the need to verify correlations between both tools [15,16,38].

Across the 18 included studies, several consistent patterns emerged. The most frequently reported landmarks were those of the nasal region (pronasale, subnasale, nasion), the labial region (labiale superius, labiale inferius, stomion, cheilion), and the mandibular region (soft tissue pogonion, gnathion, menton), reflecting the clinical relevance of these structures in orthognathic and orthodontic assessment. Angular and linear measurements were most commonly used to quantify facial symmetry, nasolabial angle, and inter-landmark distances in the vertical and transverse planes. Functionally, the included studies addressed four main clinical contexts: evaluation of soft-tissue changes following orthodontic treatment [24,35], assessment of facial asymmetry [25,33,39], characterization of nasal changes after orthognathic surgery [27], and normative anthropometric analysis [31,34]. Importantly, none of the 18 included studies incorporated artificial intelligence or automated landmark detection, representing a key evidence gap that limits the scalability of 3D facial analysis in clinical workflows and restricts it to manual scanning and measurement.

Recent studies [23,42] have explored methods to automate the capturing and analysis of 3D images, enhancing efficiency and accuracy within the process. These innovations could revolutionize clinical practice by facilitating more rapid and precise planning and personalizing treatments based on more detailed and objective data.

Finally, this study rests on several topics widely documented in the community: (1) soft tissues are poorly represented in CBCT [13,14]; (2) 3D photography is an extensively utilized tool that can be developed with easily accessible equipment [23,42]; (3) facial surgical planning significantly influences not only the execution of the surgery but also patient predisposition and acceptance of results [17,18,19]; and (4) the rising demand for substantial facial changes today requires greater preparedness in addressing these types of treatments [14,17]. Thus, a three-dimensional tool for analysis, classification, and planning of facial surgical treatment, both functional and aesthetic, represents a necessary starting point for achieving satisfactory results.

Furthermore, the search was restricted to articles published in English or Spanish, which may have excluded relevant contributions in other languages—including Chinese, Russian, and Japanese—that could enrich the evidence base for 3D facial landmark analysis.

## 5. Conclusions

Available evidence from observational studies suggests that 3D facial photography is a promising and increasingly utilized tool for morphological evaluation in orthognathic surgery and orthodontics. However, the heterogeneity of imaging systems, landmark definitions, and measurement protocols, combined with the absence of a formal risk-of-bias assessment, precludes definitive conclusions about precision or clinical utility. Key advances documented across the included studies include reliable stereophotogrammetric landmark identification, quantification of soft-tissue changes following orthodontic and orthognathic treatment, and characterization of facial asymmetry and proportionality. Remaining gaps include the absence of a validated consensus landmark set, lack of normative reference values stratified by age, sex, and ethnicity, and notably, the absence of AI-based analytical approaches in any of the 18 included studies. The 3D FullFace A framework is presented as a preliminary synthesis requiring prospective reliability and validity testing before clinical integration.

## Figures and Tables

**Table 1:** Data extraction table; author, year, type of study objectives included.

Author and Year	Study Design	N	Objective
Zhou et al. [24]	Observational, longitudinal, case series	52	Evaluate the changes in soft tissues in the nasolabial folds after orthodontic treatment through image overlap and assessment of points pre- and post-treatment.
Wu et al. [25]	Observational, case series	330	Evaluate facial symmetry through 3D overlay of facial segments defined by landmarks.
Sutherland et al. [26]	Observational, case series	78	Evaluate the relationship between facial diagnosis and the severity of apnea in children.
Silva et al. [27]	Observational, case–control study	15	Evaluate three-dimensional nasal alterations in Le Fort I advancement: linear measurements, angles, nasal indices, and volume differences.
Riml et al. [28]	Observational, case series	10	Evaluate the differences produced by varying positions during facial image acquisition.
Pucciarelli et al. [29]	Observational, clinical case presentation	1	Evaluate the efficacy of autologous fat grafting in the treatment of Parry–Romberg syndrome.
Parra et al. [30]	Observational, case series	30	Evaluate the changes in nasal morphology by comparing resting position with maximal smile.
Liu et al. [31]	Observational, case series	189	Conduct a 3D analysis of facial morphology of two different racial groups.
Launonen et al. [32]	Longitudinal observational, case series	75	Evaluate the variations in asymmetry in growth from one to three years.
Kale & Buyukcavus, [33]	Observational, case series	75	Evaluate dimensional differences between skeletal class III and pseudo class III.
Godt et al. [34]	Observational, case series	290	Evaluate the correlation between occlusal anomalies and facial analysis parameters.
Gao et al. [35]	Observational, case series	52	Evaluate changes in facial soft tissues after orthodontic treatment.
Dindaroğlu et al. [36]	Observational, case series	80	Compare the reproducibility and accuracy of measurements performed in 3D facial images versus 2D facial images.
Chu et al. [37]	Observational, case series	240	Analyze the relationship between sex and nasal morphology in Chinese individuals.
Cascos et al. [38]	Observational, case series	60	Compare the accuracy of 2D photography and 3D facial scanning for taking anthropometric measurements.
Bernini et al. [39]	Observational, case series	76	Level IV (case series, cross-sectional)
Baysal et al. [40]	Observational, case series	34	Evaluate the reliability and reproducibility in identifying facial landmarks in 3D photogrammetry.
Andrade et al. [41]	Observational, case series	30	Study of repeatability of angular and linear measurements in facial morphology analysis using stereophotogrammetry.

**Table 2:** Landmarks and measurements and their descriptions in the original article.

Author and Year	Landmarks and Measurements
Zhou et al. [24]	Me′: Soft tissue menton; Pog′: Soft tissue pogonion; B′: Soft tissue b point; A′: Soft tissue A point; SN: Subnasale; TN: Tip of nose; Ls: Highest point of upper lip; STM: Midpoint of upper and lower mouth cracks; Li: Lowest point of the lower lip; CphR(L): Crista Philtri right (and left); ChR(L): Right corner of the mouth (and left)
Wu et al. [25]	Tr (Trichion): The point located below the hairline in the midline of the forehead. En (Endocanthion): The soft tissue point located at the inner commissure of each eye fissure. Ex (Exocanthion): The soft tissue point located at the outer commissure of each eye fissure. N (nasion): The nasion of soft tissue. Sn (Subnasale): The midpoint on the nasolabial soft tissue contour between the columella crest and the upper lip. Ch (Cheilion): The point located at each labial commissure. Li (Labiale inferius): The midpoint of the vermilion line of the lower lip.
Sutherland et al. [26]	G′: Soft tissue glabela; SN: Soft tissue subnasale; GN: Soft tissue gnathion; T: Tragus; N: Soft tissue nasion; SL: Sublabiale; Me: Menton; Cer: Cervical point. Soft tissue point midway between menton and cricoid; NP: Neck plane. Inferior point of the anterior neck plane.
Silva et al. [27]	Midline landmarks: Tr (trichion); G (glabella); N (nasion); Prn (pronasale); C (columella); Sn (subnasale); Ls (labiale superius); Sto (stomion); Li (labiale inferius); Sl (sublabiale); Pg (pogonion); Me (menton); Ex (exocanthion); En (endocanthion); Os (orbitalesuperius); Or (orbitale); Ft (frontotemporale); Chk (cheek); Zy (zygion); Tr (traguion); Al (alare); Ac (nasal alar crest); Cphr (crista philtri); Ch (cheilion); and Go (gonion).
Riml et al. [28]	Front; Nasal dorsum:Upper lip:Chin:Jaw angle; Nasolabial angle
Pucciarelli et al. [29]	ac, nasal alarcrest; al, alare; ch, cheilion; chp, crista philtri; en, endocanthion; ex, exocanthion; gl, glabella; gn, gnathion; go, gonion; n, nasion; or, orbitale; os, orbitale superius; prn, pronasale; sa, superaurale; sl, sublabiale; sn, subnasale; sto, stomion; t, tragion; tr, trichion; zy, zygion.
Parra et al. [30]	Alar base length; Nasolabial angle
Liu et al. [31]	Nasion: Deepest point of nasal bridge; Endocanthion right (left): Inner commissure of the eye fissure. Exocanthion right (left): Outer commissure of the eye fissure; Orbitale right (left): Lowest point on the lower margin of the orbit; Pronasale: Most protruded point of the apex nasi; Subnasale: Midpoint of the angle at the columella base; Alare right (left): Most lateral point on the right (or left) alar contour; Subalare right (left): Lower limit of the right (or left) alar base; Labium superius: Midpoint of the upper vermilion line; Cristaphiltri right (left): Point on the right (left) elevated margins of the philtrum just above the vermilion line; Stomion: imaginary point at the crossing of the vertical facial midline and the horizontal labial fissure; Cheilon right (left): Point located at the right (left) labial commissure; Pogonion: Most anterior midpoint of the chin; Gnathion: Lowest median landmark on the lower border of the mandible; Zygion right (left): Most protrusive point of the right (left) zygomatic arch.
Launonen et al. [32]	Glabella (g); Nasion (n); Endocanthion (en); Exocanthion (ex); Pulpabrale superius (ps); Pulpabrale inferius (pi); Pronasale (prn); Subnasale (sn); Alare (al); Labiale superius (ls); Labiale inferius (li); Christa philtra (cph); Cheilion (ch); Pogonion (pg); and Tragion (Tr).
Kale & Buyukcavus, [33]	Nasion; Glabella; Exocanthion right (left); Endocanthion right (left); Pronasale; Subnasale; Alar right (left); Crista Philtri right (left); Labiale superius; [20,21,32,34] lion right (left); Stomion; Labiale inferius; Sublabiale; Pogonion; Gnathion; Menton; Tragus right (left); Gonion right (left); Tragion.
Godt et al. [34]	Glabella; Nasion; Pronasale; Zygomatic right (left); subnasale; Columella; Alar right (left); Supralabiale; Labiale Superius; Stomion; Labiale inferius; sublabiale; Pogonion; Menton; Eurion.
Gao et al. [35]	Nasal Ala; Soft tissue b point; Cheilion; Endocanthion; Exocanthion; Frontotemporal point; Glabella; Soft tissue gonion; Labrale inferior; Labrale superior; Soft tissue menton; Soft tissue nasion; pronasale; Soft tissue pogonion; Sobnasale; Trichion; Tragus; Upper Lip Point; Zygomatic point.
Dindaroğlu et al. [36]	Tragus; Exocanthion; Endocanthion; Glabella; Nasion; Pronasal; Columella; Alare; Subnasal; Labium superior; Stomion; Labium inferior; Supramental; Menton; chellion.
Chu et al. [37]	Soft Nasion, Pronasle, Subnasale, Ala (Right and Left), Labial superius.
Cascos et al. [38]	Glabella; Internal Endocanthion of the right (And left eye); external exocanthion of the right eye (Ans left eye); Subnasal; Right Chelion; Left Chelion; and Menton (Me).
Bernini et al. [39]	ND
Baysal et al. [40]	Nasion; Glabella; Endocantion (right and left); Pronasale: Subnasale; Philtrum (right and left); Pogonion; Exocanthion (right and left); Alare (right and left); Cheilion (right and left); Labiale superior; Labiale inferior; Zygion (right and left).
Andrade et al. [41]	Nasofrontal angle (Glabella-Nasion-Pronasale); Nasolabial angle (Columella-subnasale-labiale superius); Mentolabial angle (labiale inferius-Sublabiale-Pogonium); Full facial convexity angle (Glabella-Pronasale-Pogonium); Facial convexity angle (Glabela-Subnasale-Pogonium); Nasal angle (Nasion-Tragus-Pronasale); Maxillary angle (Pronasale-Tragus-labiale superius); Mandibular angle (Labiale superius-Tragus-Pogonium); Maxilofacial angle (Labiale superius-Nasion-Pogonium); MFH (Glabella-Subnasale); LFH (Subnasale-Menton).

**Table 3:** Linear measurements (in mm) of the FullFace facial analysis; analysis area; name of the measurement; selected points; and objective and description of the measurement.

Area	Measurement	Points	Objective/Description
**Facial Vertical Ratio**	Facial Thirds	- Tr-Gl; - Gl-Sn; - Sn-Mn	To evaluate vertical facial proportion divided into thirds. - Upper third: Distance between points Tr and Gl; - Middle third: Distance between points Gl and Sn; - Lower third: Distance between points Sn and Mn.
	Middle Third	- Gl-P; - P-Sn	To evaluate vertical proportion of the middle third. - Upper segment of middle third: Vertical distance from glabella to the horizontal midpoint between points P right and left; - Lower segment of middle third: Vertical distance from the horizontal midpoint of points P right and left to Sn.
	Lower Third	- Sn-St; - St-Mn	To evaluate vertical proportion of the lower third. - Upper segment of lower third: Vertical distance between points Sn and St; - Lower segment of lower third: Vertical distance between points St and Mn.
**Horizontal Facial Ratio**	Facial Fifths	- Zy-Ex (right and left); - Ex-Ed (right and left); - Edd-Edi (right and left);	To evaluate facial proportionality in the horizontal plane. - Middle segment: Horizontal distance between points Ed right and left; - Medial right segment (and left): Horizontal distance between points Ed and Ex right and left; - Lateral segments (right and left): Horizontal distance between points Ex and Zy right and left.
**Middle Third Analysis**	Eye Analysis	- Pd-Pi - Ed-Ex (right and left) - PS-PI (right and left) - P-midline (right and left) - P-Zy (right and left)	To evaluate the shape of the eye and its spatial disposition among the face. - Interpupillary distance: Horizontal distance between points P right and left; - Width of the eye: Horizontal distance between points Ed and Ex right and left; - Height of the eye: Vertical distance between points PS and PI; - Horizontal position of the eye relative to the midline: Distance from point P (right and left) to the sagittal midline; - Horizontal position of the eye relative to the face’s outer edge: Distance between point P right (or left) and point Zy right (and left).
	Nasal Analysis	- Ald-Ali - Sn-Pn - Na-Pn	To evaluate nasal morphology. - Width of the nasal wing: Represented by horizontal distance between points Al right and left; - Nasal length (anteroposterior): Anteroposterior distance from Sn to Pn; - Three-dimensional distance between points Na and Pn.
**Lower Facial Third Analysis**	Lip Analysis	- CLd-CLi - LS-St - St-LI - LS-LI - Cphd-Cphi - Cph-LS (right and left)	To evaluate lip morphology. - Width of the lips: Distance between points CL right and left; - Height of the upper lip: Vertical distance between points LS and St; - Height of the lower lip: Vertical distance between points St and LI; - Height of the lips: Vertical distance between points LS and LI; - Lip filter: Horizontal distance between points Cph right and left; - Height of the filter: Vertical distance between points Cph and LS (right and left).
	Chin Analysis	- B′-Mn - B′-Pog′	To evaluate chin morphology. - Chin height: Vertical distance from points B′ to Mn; - Chin width (anteroposterior): Anteroposterior distance from points B′ to Pog′.
	Mandible Analysis	- God-Goi - Go-Gn (right and left) - Go-Gn (right and left)	To evaluate mandibular morphology - Width of the mandible: Horizontal distance between points Go right and left; - Mandible base: Linear distance between points Go and Gn; - Mandible width: Anteroposterior distance between points Go and Gn.

**Table 4:** Angular measurements of the FullFace facial analysis; analysis area; analyzed structures; selected points; and objective and description of the measurement.

Area	Measurement	Objective/Description
**Middle Third Analysis**	Eye Analysis	To evaluate the morphology and spatial positioning of the eyes. - Eye inclination: Angle formed by the intersection of the line Ed and Ex and the horizontal plane; - Spatial disposition of the eye: Angle formed by the right and left pupil points and point Sn; - Medial eye angle: formed by points PS, Ed, and PI; - Lateral eye angle: formed by points PS, Ex, and PI; - Upper eye angle: formed by points Ex, PS, and Ed; - Lower eye angle: formed by points Ex, PI, and Ed; - Eye–nose spatial positioning: Angle formed by right pupil point, Sn, and left pupil point.
	Nasal Analysis	To evaluate the morphology and spatial disposition of the nose. - Nasal width angle: Angle formed by points Al right and left and point Na; - Nasal base angle: Formed by points Al right and left and point Sn; - Nose tip angle (coronal view): Formed by points Al right and left and point Pn. - Fronto-nasal angle: Formed by the intersection of points Gl, Na, and Pn; - Nose tip angle (sagittal view): Formed by the intersection of points Na, Pn, and Sn. - Nasolabial angle: formed by points LS, Sn, and Pn.
**Lower Third Analysis**	Lip Analysis	To evaluate the morphology and spatial disposition of the lips. - Labial commissure angle: Formed by the intersection of points LS, CL (right or left), and LI; - Upper lip angle: Formed by points CL right and left and point LS; - Lower lip angle: Formed by points CL right and left and point LI; - Lip inclination angle: Angle formed by the line CL (right and left) and the horizontal plane; - Lateral crest angle: Formed by points Cph (right or left), CL (right or left), and LI.
	Chin Analysis	To evaluate the spatial positioning of the chin. - Lateral position of the chin: Formed by the line Mn-LI and the median sagittal plane; - Anteroposterior position of the chin: Formed by angles Tr, Sn, and Pog′.
**Lateral Face Analysis**	Eye Analysis	To evaluate the anteroposterior positioning of the eyes. - Eye AP: Tr-SN-P (right and left).
	Nasal Analysis	To evaluate the anteroposterior position of the nose. - Nose AP: Union of points Tr-Sn-Pn.
	Maxilla Analysis	To evaluate the anteroposterior position of the maxilla. - Maxilla AP: Union of points Tr-Sn-A′.
	Mandible Analysis	To evaluate the morphology and positioning of the mandible. - Mandibular inclination: Formed by the line Tr-Sn and the basilar border of the mandible; - Anterior mandible: Formed by the union of points Go right and left and point Mn; - Anteroposterior position of the mandible: Union of points Tr-Na-B′; - Anteroposterior position of the chin: Union of points Tr-Sn-Pog′.

## Data Availability

No new data were created or analyzed in this study.

## Supplementary Materials

The following supporting information can be downloaded at: https://www.mdpi.com/article/10.3390/jcm15156091/s1, Table S1: Selected Midline Landmarks. Name, abbreviation, and description of all the points selected on the facial midline, as well as the articles in which they were used; Table S2: Bilateral points. Name, abbreviation, and description of all the points selected on both sides of the face, along with the articles in which they were used.
