# Supplementary material for: Anatomical References for the Study of 3D Facial Photographs: A Scoping Review and Proposed Preliminary Framework for Comprehensive Facial Evaluation
Source: J Clin Med. 2026 Aug 5;15(15):6091. doi: 10.3390/jcm15156091 (PMC13466362; doi:10.3390/jcm15156091)
Supplement: Supplementary file 1 [file jcm-15-06091-s001.zip › jcm-4392824-supplementary.pdf]

TABLES

Table S1. Selected Midline Landmarks. Name, abbreviation, and description of all the points selected on the facial midline, as well as the articles in which they were used.

Name	Abbreviation	Description
Trichion	Tri	point located below the hairline in the midline of the forehead. [21,23]
Glabela	Gl	The glabela is a median elevation between the two superciliary arches. [22, 23, 28–30, 34, 36, 37]
Nasion	Na	Deepest point of the concavity formed between the eyes in the frontonasal area.[27–29, 31, 33, 36, 37]
Pronasal	Pn	Point located at the tip of the nose; most anterior point of the nasal contour. [20, 23–29, 31, 32, 36, 37]
Subnasal	Sn	Deepest and median point of the nasal base, between the upper lip and the columella.[20, 21, 23–29, 31–34, 36, 37]
Soft Tissue A′	A′	Deepest point of the anterior concavity located between the upper lip and the nose. [20]
Labiale Superior	LS	Midpoint of the upper lip contour. [23, 25, 27, 28]
Stomion	St	Midpoint located on the line between the upper and lower lips. [20, 23, 25, 27, 29, 32]
Labiale Inferior	LI	Midpoint of the lower lip contour. [20–23, 25, 28–30, 32, 36, 37]
Soft Tissue B′	B′	Deepest point located in the anterior concavity between the pogonion and the lower lip. [20, 31]
Soft Tissue Pogonion	Pog′	Most anterior point of the chin. [23, 28, 29, 31, 36, 37]
Gnation	Gn	Most anterior and inferior point of the chin, typically located between the pogonion and the menton. [21, 22,27,32)]
Menton	Mn	Lowest point of the chin. [20, 22, 23, 29, 30, 32, 34]

Table S2. Bilateral points. Name, abbreviation, and description of all the points selected on both sides of the face, along with the articles in which they were used.

Name	Abbreviation	Description
Zygion	Zy	Most lateral point of the zygomatic arch. [23, 25, 31, 36]
Gonion	Go	Point situated at the lowest, posterior, and lateral angle of the external mandibular angle. [20, 23, 25, 29, 31]

Edocanthion	Ed	Point where the inner ends of the upper and lower eyelids meet. [21, 23, 25, 27–29, 31, 32, 34, 36]
Exocanthion	Ex	Point where the outer ends of the upper and lower eyelids meet. [21, 23, 25, 27–29, 31, 32, 34, 36]
Superior Palpebral	PS	Landamark located at the highest point of the upper eyelid.
Inferior Palpebral	PI	Landmark located at the lowest point of the lower eyelid.
Orbitale	Or	Lowest point of the lower margin of the orbit. [23, 25, 27]
Pupilar	P	Point located at the center of the pupil.
Tragus	T	Point located at the most posterior and median part of the anatomical tragus. [22, 29, 31, 32, 37]
Alar	Al	Point located at the most lateral part of the wing of the nose. [23, 25–28, 30, 32, 36]
Labial Commissure	CL	Point located at the most lateral vertex between the upper and lower lips. [18, 20, 24, 27, 30, 34]
Cristra philtri	Cph	Point located at the junction of the lateral crest of the filter and the vermilion of the upper lip. [20, 23, 25, 27, 29]
